# Kinematics and Aerodynamics of Dragonflies (*Pantala flavescens*, Libellulidae) in Climbing Flight

**DOI:** 10.3389/fbioe.2022.795063

**Published:** 2022-03-16

**Authors:** Liansong Peng, Tianyu Pan, Mengzong Zheng, Shiying Song, Guanting Su, Qiushi Li

**Affiliations:** ^1^ School of Energy and Power Engineering, Beihang University, Beijing, China; ^2^ Reserach Institute of Aero-Engine, Beihang University, Beijing, China; ^3^ Key Laboratory of Fluid and Power Machinery, Ministry of Education, Xihua University, Chengdu, China

**Keywords:** dragonfly, climbing, kinematics, aerodynamics, wing

## Abstract

This study presents a detailed analysis of dragonflies’ climbing flight by integratinghigh-speed photogrammetry, three-dimensional reconstruction, and computational fluid dynamics. In this study, a dragonfly’s climbing flight is captured by two high-speed cameras with orthogonal optical axes. Through feature point matching and three-dimensional reconstruction, the body kinematics and wing kinematics of 22 dragonflies in climbing flight are accurately captured. Experimental results show that the climbing angles (*η*) are distributed from 10° to 80° and are concentrated within two ranges, 60°–70° (36%) and 20°–30° (32%), which are defined as large angle climb (LAC) and small angle climb (SAC), respectively. In order to study the aerodynamic mechanism of the climbing flight based on the biological observation results, the kinematic parameters of the dragonfly during LAC and SAC are selected for analysis and numerical simulation. The results show that the climbing angle *η* and wing kinematics are related. There are considerable differences in wing kinematics during climbing with different *η*, while the wing kinematics are unchanged during climbing with similar *η*. With the increase in *η*, the phase difference (*λ*) between the forewing and the hind wing decreases and the amplitude of the positional angle (*θ*_mean_) of the hind wing increases, while *θ*_mean_ of the forewing remains almost unchanged. Through numerical simulation of LAC and SAC, it can be found that during the climb with different *η*, the different wing kinematics have a significant influence on aerodynamic performance. During SAC, the increase in *λ* and the decrease in *θ*_mean_ of the hind wing weaken the aerodynamic disturbance of the forewing by the vortex wing of the hind wing, thus improving the flight efficiency.

## Introduction

With the development and application of micro air vehicles (MAVs), the study of insect flight mechanisms has attracted more and more attention (Sun, 2014; Floreano and Wood, 2015; Bomphrey et al., 2016). Inspired by flying insects and other flying animals, researchers attempted to apply efficient flapping wing mechanisms to MAVs (Heers et al., 2018; Koizumi et al., 2021; Murayama et al., 2021). Dragonflies are aerial predators with outstanding flight abilities. They possess unique flight characteristics and aerodynamic advantages. Dragonflies are rare insects with two pairs of wings that flap independently (Alexander, 1984). They can hover (Ellington, 1984), turn 90°–180° in two or three wing beats (Li and Dong, 2017), glide (Wakeling and Ellington, 1997a), and produce total aerodynamic force equal to ∼4.3 times their own body weight (Su et al., 2020). Therefore, researchers are interested in their unique flapping characteristics and excellent flying skills, and hope that studying the aerodynamic characteristics of dragonflies can provide guidance for the optimization of MAV (Liu et al., 2021a; Liu et al., 2021b). The accurate description of flapping kinematics and the investigation of flapping aerodynamics under various kinds of dragonfly flight modes (Wakeling. and Ellington, 1997a; Wang et al., 2003) provide an important data basis for the study of flapping flight mechanisms and bionic aerodynamics. With inbuilt characteristics of a phase difference between the forewing and the hind wing, independent control of each wing, and a drag-based system in hovering flight, the dragonfly has the most stable hovering capability and can switch flight modes without altering postures.

The wing kinematics of dragonfly-like MAVs are based on the real flapping of dragonflies (Du and Xu, 2015; Takahashi et al., 2016; Yousaf et al., 2020). The kinematics of dragonfly wings vary in different flight modes, so it is necessary to match the flight mode with flapping kinematics to provide a data basis for motion control of dragonfly-like MAVs. In order to accurately obtain the kinematic parameters of dragonflies under various kinds of flight modes, researchers have employed different experimental methods. In 1997, Wakeling and Ellington (1997a), Wakeling and Ellington (1997b), and Wakeling and Ellington (1997c) used two high-speed cameras with perpendicular optical axes to capture the kinematic parameters of dragonflies and damselflies during gliding and free forward flight. In 2002, Wang et al. (2003) employed the projection comb fringe interpolation method to measure the kinematic parameters of dragonflies and wing deformation during forward flight and turning. In 2008, Cheng et al. (2008) measured the wing deformation of dragonflies during turning using the projection grating method. In 2017, Li and Dong (2017) photographed free-flying dragonflies with three cameras and proposed that the ratio of arch deformation to chord length to represent the arch deformation of wings.

By summarizing the observation results of free-flying dragonflies (Azuma et al., 1985; Somps and Luttges, 1985; Saharon and Luttges, 1989; Wakeling and Ellington, 1997c), consensus has been reached that the dragonfly employs counter-stroking (*λ* = 180°) when hovering, phased stroking (*λ* = 90°) when flying fast forward, and synchronized stroking (*λ* = 0°) when maneuvering. These observations provide a theoretical basis for the motion control of dragonfly-like MAVs. However, as a typical flying mode of dragonflies, especially in emergency escape or prey capture, systematic study on climbing has rarely been conducted. Dragonflies’ climbing flights were found in Azuma et al. (1985) and Wakeling and Ellington (1997c). The climbing angle (*η*) was 40° in Azuma’s observation and ranged from 11° to 81° in Wakeling’s observation. Both of them lacked detailed information on wing kinematics, and due to the small amount of flight samples, the distribution law of *η* cannot be statistically analyzed. So, how do the kinematic parameters of the body and wings change under different *η*, and what are the effects of these parameters on aerodynamic performance?

In this study, the kinematics and the aerodynamic mechanism of dragonfly climbing were investigated by analyzing the biological observation data and numerical simulation. Dragonflies were photographed by two high-speed cameras with orthogonal optical axes. Detailed body kinematics and wings kinematics during the climbing flight were obtained. According to the experimental results, it was found that the dragonfly’s climbing angle ranged from 10° to 80°. Two typical climbing statuses of dragonflies were selected for analysis in this study: the LAC with *η* between 60° and 70°, and the SAC with *η* between 20° and 30°. (The probability of occurrence in experimental observations is 36 and 32%, respectively.) The kinematic parameters of the body and wings during LAC and SAC were analyzed and compared by using feature point matching and a three-dimensional reconstruction method. The aerodynamic characteristics during LAC and SAC were compared and studied by using a numerical simulation method, which provided guidance for the exploration of the bionic aerodynamic theory and the engineering application of MAV. The kinematics of wings extracted from LAC and SAC provides the data basis for flight control of tandem-wing MAVs.

## Materials and Methods

### Experimental Observation

**FIGURE 1:**
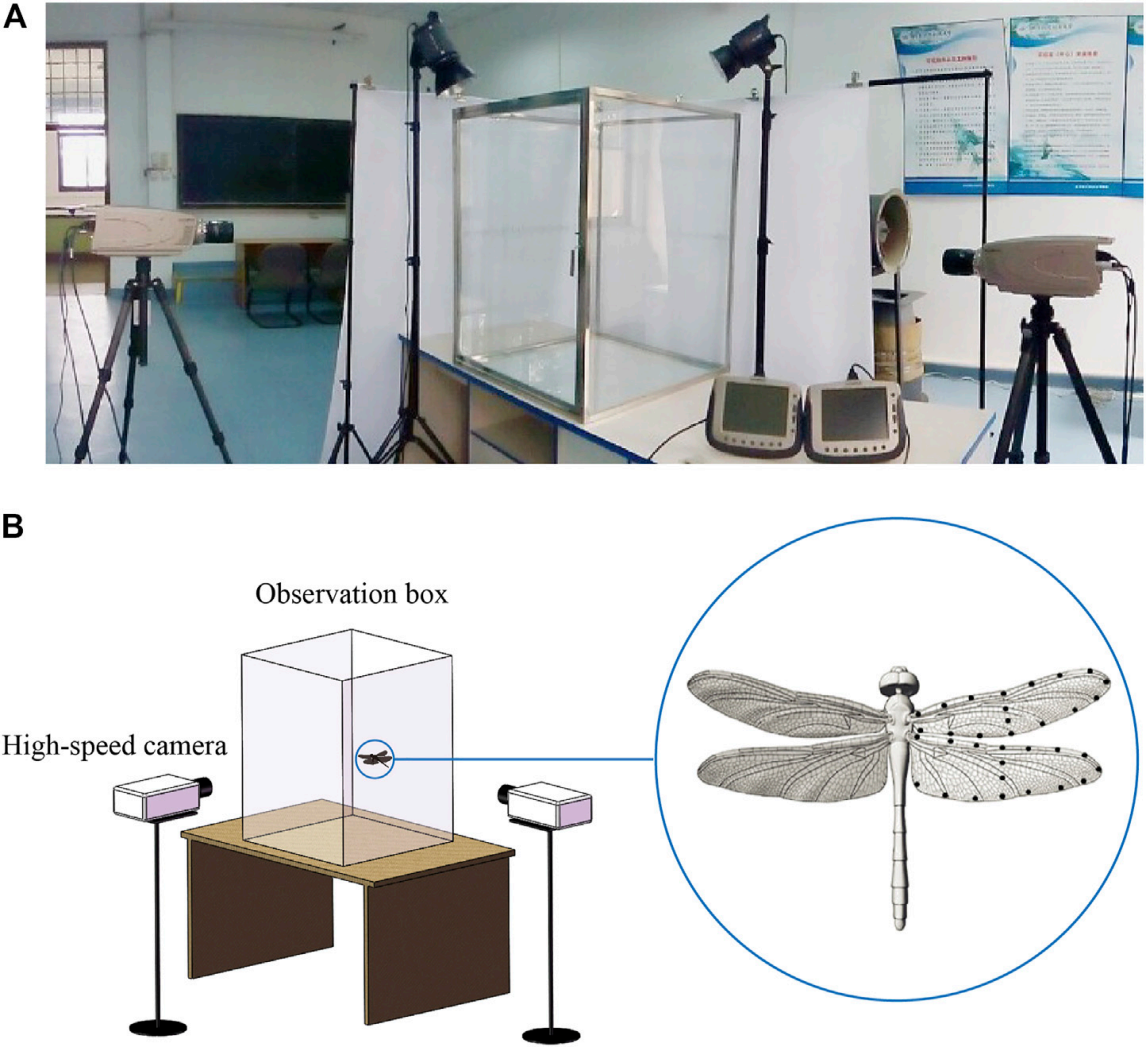
**(A)** Experimental facilities. **(B)** Line drawing of experimental facilities and feature points distribution.

*Pantala flavescens* belongs to a widely distributed dragonfly family (Libellulidae) and is considered to be the most widespread dragonfly on the planet. The dragonflies measured in this experiment were collected from a pond at the main campus of Beihang University (Beijing, Peoples Republic of China). The dragonflies were then transferred to the indoor laboratory in a dark container and were used in experiments on the day of capture. Following hypothermic anesthetization of individual dragonflies, the morphological parameters of their bodies and wings were measured, and black feature points were marked on the wings. As shown in Figure 1A, two orthogonally arranged high-speed cameras (Olympus i-SPEED TR, 1,000 frames^−1^, shutter speed 1 ms, resolution 1,280 × 1,024 pixels) were used to film the climbing flight of dragonflies within a glass observation box (volume of 0.6 m^3^; 0.78 m × 0.78 m × 1.0 m). In order to prevent the visual system of the dragonflies from being affected by the experimental equipment and the surrounding environment during flight, the observation box was made of transparent glass, and white curtains were used to separate the dragonflies from the surrounding environment without affecting the camera’s vision. The distribution of feature points is shown in Figure 1B for three-dimensional reconstruction. The experimental configuration and three-dimensional reconstruction method are described in detail in Li et al. (2018). Detailed experimental methods are described in electronic Supplementary Material S3. The dragonflies recovered from anesthesia were placed on the bottom platform of the observation box, which was 0.3 m below the vertical coverage area of the two cameras. When the dragonflies climbed into the vertical coverage area, the trigger switch of the cameras was pressed to get the photo sequences of dragonfly flight. Each dragonfly made one to three flight attempts and then was released.

### Numerical Simulation

The fluid simulation was carried out using the commercial computational fluid dynamic (CFD) solver XFlow 2019, which solves the three-dimensional Boltzmann’s transport equation based on the lattice Boltzmann method (LBM). The LBM works on a spatial discretization named lattice, consisting of a Cartesian distribution of discrete points with a discrete set of velocity directions ***e***_***i***_ (*i* = 1, …, *b*). In this method, the continuum is regarded as discrete particles on a lattice. Particles move on the lattice and collide with each other according to specific rules. Through the statistics of particles and the motion characteristics, the macroscopic characteristics of fluid can be obtained.

LBM schemes were classified as a function of the spatial dimensions d and the number of velocity distribution functions b, resulting in the notation DdQb. In the present method, the 27-velocity model (D3Q27), together with a central moment collision operator, was adopted to enhance the numerical stability. The governing equation of the flow field in continuum space is as follows:

$$\frac{\partial f_{i}\left(R,\;t\right)}{\partial t}+e_{i}\nabla f_{i}\left(R,\;t\right)=\Omega_{i},i=1,...,b,$$ (1)

where *f*_*i*_ *(R, t)* is the distribution function for particles with velocity ***e***_***i***_ at position *R* and time *t*, and ***Ω***_***i***_ is the collision operator that computes a post-collision state conserving mass and linear momentum. Eq. 1 is discretized on the lattice as follows:

$$f_{i}\left(R+e_{i}\right)=f_{i}\left(R,t\right)+\Omega_{i}\left(f_{1},...,f_{b}\right),\;i=1,...b.$$ (2)

The Cartesian lattice is used and the meshing process is greatly simplified, which makes the lattice Boltzmann method (LBM) have advantages over conventional numerical methods in solving moving boundary conditions with complex surfaces.

A detailed description of this solver is given in Peng et al. (2021), so only brief introductions are provided here. In the current study, simulations were conducted on a computational domain with dimensions of 70*c* × 40*c* × 30*c* in terms of average wing chord length (*c*). To resolve the near-wake vortex structures, the unsteady flow near the flapping wings was computed with a fine resolution of 0.01*c* and 200 time steps per cycle, and the total number of computational grid points was about 10.2 million. When further refinements to the domain and time step were applied, the variation in the mean total lift was less than 0.5%. The validation of the numerical method and detailed settings of the simulation are introduced in electronic Supplementary Material S1, S2, respectively.

### Definition of Kinematic Parameters

**FIGURE 2:**
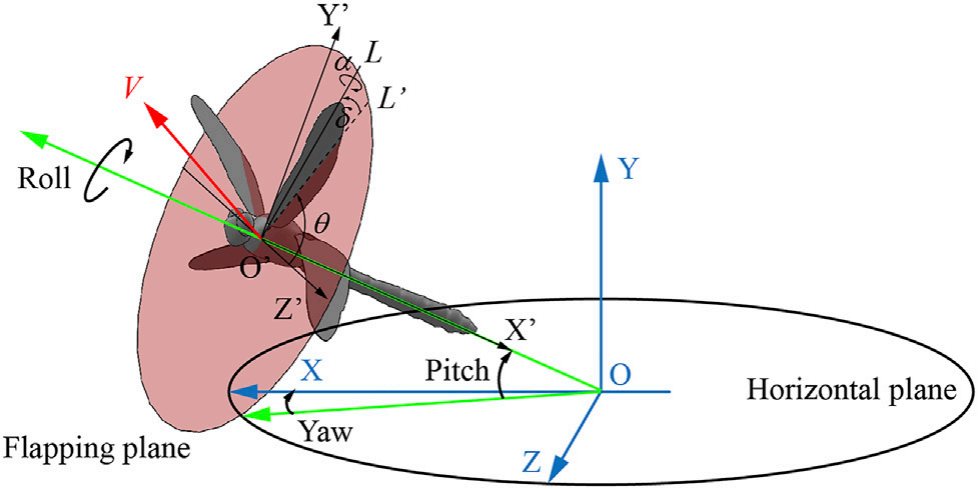
Definition of kinematic parameters.

As shown in Figure 2, OXYZ is an inertial frame with the X and Z axes in the horizontal plane, and O’X’Y’Z’ is a relative coordinate system, where point O′ is the center of mass. Phase difference *λ* is an important kinematic parameter for the interactions, which is defined as the phase lag between the forewing and the hind wing. We defined the phase difference *λ* as positive when the hind wing leads the forewing and negative when the forewing leads the hind wing. The climbing angle *η* is defined as the angle between the direction of average flight velocity *V* during climbing and the horizontal plane, and the pitch angle *ε* is defined as the angle between the dragonfly’s body longitudinal axis and the horizontal plane. The body posture of a dragonfly in flight can be represented by three Euler angles: pitch angle, yaw angle, and roll angle. Since the yaw angle and roll angle were approximately zero or 180° during the climb, the body posture can be described by *ε*. By analyzing the photo sequence, the trajectory of wing tip mark points during the stroke was obtained. Based on these points, a line can be determined by using the linear regression method. As a result, the flapping plane is defined based on the wing tip line and the root of the wing. The flapping plane angle *β*_H_ is the angle between the flapping plane and the horizontal plane. *L* is the line joining the wing base and wing tip, and *L*’ is the projection of *L* onto the flapping plane. The position of the wing with respect to the flapping plane was determined by three Euler angles: the positional angle (*θ*), rotational angle (*α*), and deviation angle (*δ*), where *θ* is defined as the angle between the Z’-axis and *L*’, *α* is defined as the angle between the wing plane and the flapping plane, and *δ* is defined as the angle between *L* and *L*’.

## Results

### Experimental Observation Results of Dragonfly Climbing

#### Climbing Habits of Dragonflies

**FIGURE 3:**
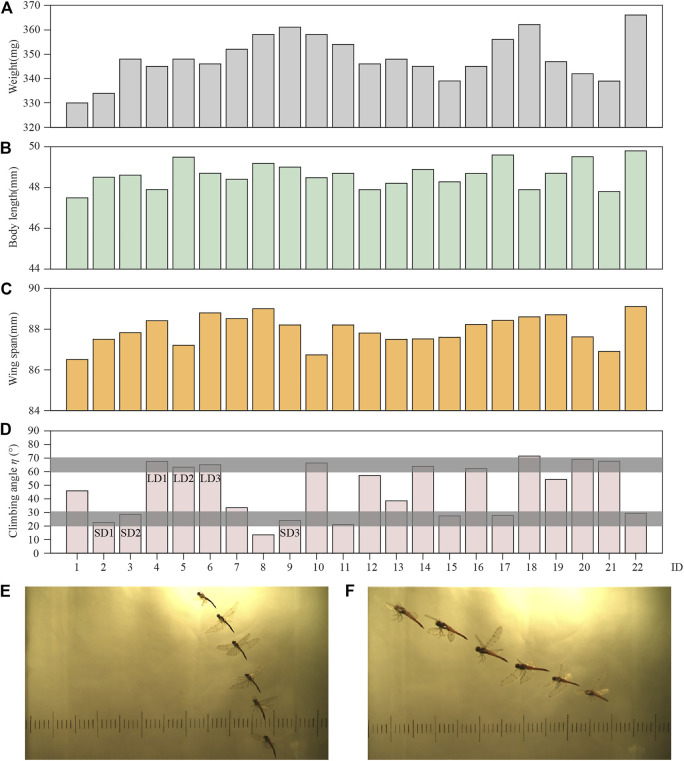
Data of **(A)** weight; **(B)** body length; **(C)** wing span; **(D)** climbing angle; **(E)** body postures of LD1; and **(F)** body postures of SD1.

To ensure the validity of the experimental data, the photo sequences of climbing flight satisfied the following conditions: the flight process contains more than two wingbeats; the flight trajectory was approximately a straight climb; and dragonflies’ body rotation was negligible. Under these conditions, 22 climbing flight sequences were analyzed, with each sequence from a different dragonfly. The mass, body length, and wingspan of the dragonflies are shown in Figures 3A–C. Due to the negligible difference in body size of the dragonflies employed in the experiment, the influence of this factor on the experiment was ignored.

These 22 climbing flight sequences were statistically analyzed. The results show that the dragonflies climb with *η* between 10 and 80°. According to the distribution of *η* in Figure 3D, the climbing angle is concentrated in two ranges, 60–70° and 20–30° (the grey boxes), which account for 36 and 32% of the climbs, respectively. Since dragonflies have a wide range of climb angles, in order to perform an efficient and detailed analysis of the climbing flight, the next step of research will focus on the two ranges, which are defined as large angle climb (LAC) and small angle climb (SAC).

In this study, three groups of experimental data of LAC and SAC were selected for analysis, respectively. For these six cases [LAC cases marked as LD1 (ID = 4), LD2 (ID = 5), LD3 (ID = 6) in Figure 3D, and SAC cases marked as SD1 (ID = 2), SD2 (ID = 3), SD3 (ID = 9) in Figure 3D], the kinematic parameters of the body and wing are given in Table 1. *θ*_mean_ and *α*_mean_ are the amplitude of the positional angle and rotational angle, respectively. *θ*_min_ and *α*_min_ are the minimum positional angle and minimum rotational angle, respectively. *θ*_max_ and *α*_max_ are the maximum positional angle and maximum rotational angle, respectively. According to the data in Table 1, the wing kinematics of the three individuals in LAC cases were almost unchanged, and a similar situation also occurred in SAC cases. Therefore, the influence of body mass variation between individuals on wing kinematics can be ignored. However, there were considerable differences between the average wing kinematics of LAC cases and SAC cases, so the climbing angle was considered to be an important factor affecting wing kinematics.

**TABLE 1:** Kinematic parameters during SAC and LAC (FW for forewing and HW for hind wing).

	ID	f (Hz)		η	V	$\beta_{body}$		$\theta_{\text{mean}}$		$\theta_{\min}$		$\alpha_{\text{max}}$		$\alpha_{\text{mean}}$		$\alpha_{\min}$		$\alpha_{\max}$		λ
		FW	HW			FW	HW	FW	HW	FW	HW	FW	HW	FW	HW	FW	HW	FW	HW	
SAC	SD1	27.2	27.4	22.4	1.3	64	67	62	36	−9	−4	53	32	90	97	−39	−40	51	57	102
	SD2	26.7	26.8	28.2	1.3	63	68	59	38	−12	−1	47	37	101	106	−42	−41	59	65	104
	SD3	26.8	26.8	23.9	1.3	68	71	63	39	−11	−10	52	29	87	97	−35	v43	52	54	105
	Mean	**26.9**	**27.0**	**24.8**	**1.3**	**65.0**	**68.7**	**61.3**	**37.7**	**−10.7**	**−5.0**	**50.7**	**32.7**	**92.7**	**100.0**	**−38.7**	**−41.3**	**54.0**	**58.7**	**103.7**
LAC	LD1	28.5	28.4	67.5	1.5	65	66	65	62	−15	−12	50	48	94	99	−45	−44	49	55	77
	LD2	28.8	28.7	63.7	1.5	67	69	63	59	−20	−18	43	41	79	90	−33	−40	46	50	78
	LD3	29.1	29.0	66.2	1.5	62	65	66	64	−18	−18	48	46	101	103	−55	−44	46	59	75
	Mean	**28.8**	**28.7**	**65.8**	**1.5**	**64.7**	**66.7**	**64.7**	**61.7**	**−17.7**	**−16.0**	**47.0**	**45.0**	**91.3**	**97.3**	**−44.3**	**−42.7**	**47.0**	**54.7**	**76.7**

Bold values in the row of “Mean” are the mean values of parameters of the three individuals.

It can be seen that during LAC or SAC, the kinematic parameters were almost the same. Therefore, it can be concluded that the kinematic parameters remain similar when *η* is kept within a certain range. Therefore, the experimental data of two individuals (LD1 and SD1) were further analyzed to determine the climbing laws of dragonflies during LAC and SAC. LD1 was the fourth individual observed in the experiment, with a mass of 345 mg and a climbing angle of 67.5°. SD1 was the second observed individual with a mass of 334 mg and a climbing angle of 22.4°. As can be seen from Table 1, for LD1, the flapping frequency *f* was about 28.5 Hz and *λ* was about 77°; for SD1, *f* was about 27.2 Hz and *λ* was about 102°. For LD1, *θ*_mean_ of both the forewing and the hind wing was more than 60°. For SD1, *θ*_mean_ of the forewing was 61.3°, while *θ*_mean_ of the hind wing was only 36°. Therefore, it can be concluded that with the increase in *η*, *λ* between the forewing and the hind wing decreases and *θ*_mean_ of the hind wing increases, while *θ*_mean_ of the forewing remains almost unchanged.

#### Kinematic Parameters of Body Posture During LAC and SAC

**FIGURE 4:**
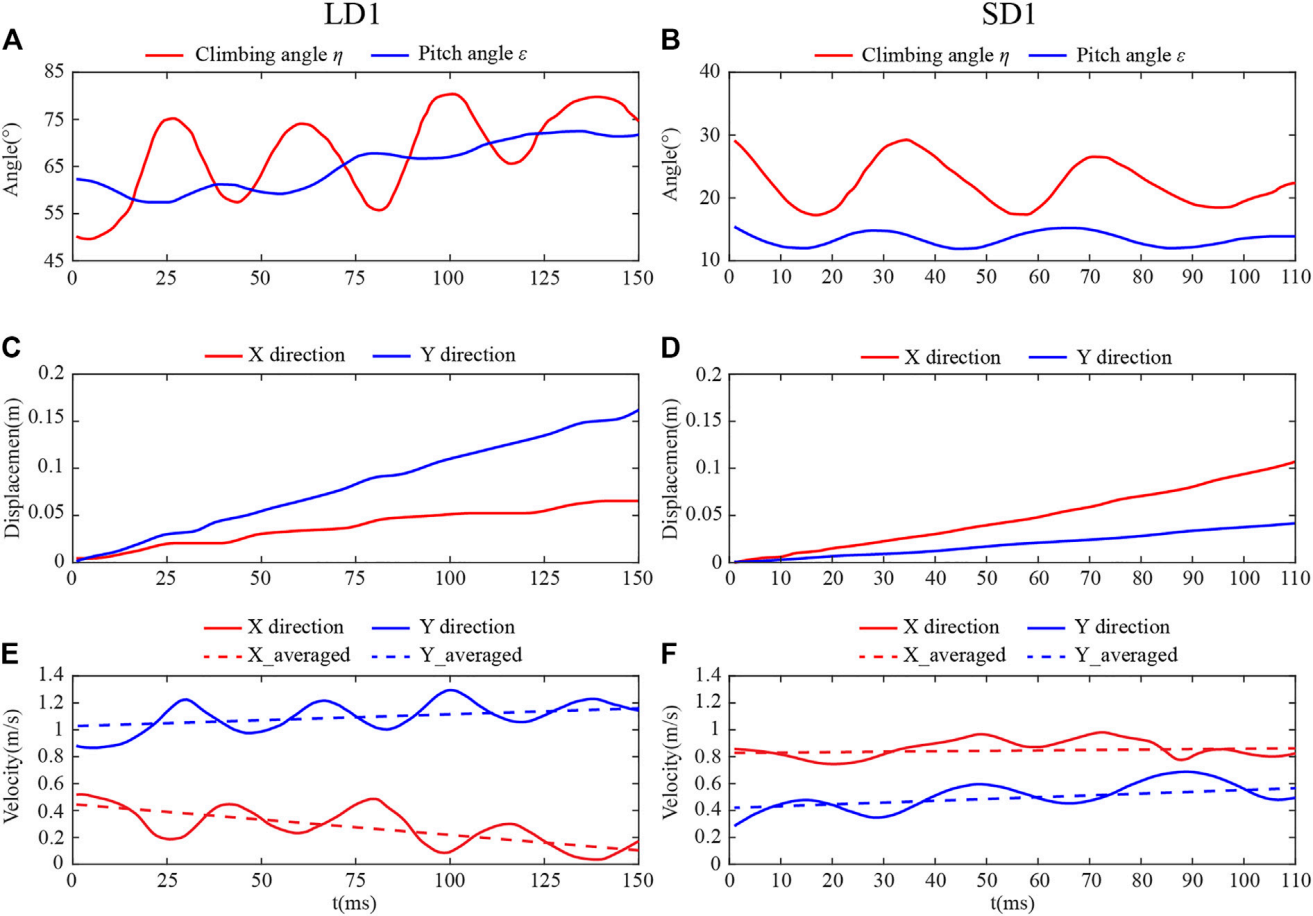
Body posture angles for **(A)** LD1 and **(B)** SD1. Flight displacement of **(C)** LD1 and **(D)** SD1. Flight velocity of **(E)** LD1 and **(F)** SD1.

Figures 3E, F show the body postures of LD1 and SD1 during climbing, respectively. The photos were taken from one of the two cameras, and it can be seen that the body posture of the dragonfly was significantly different with different *η*. The time courses of *η* and *ε* of LD1 and SD1 are shown in Figures 4A,B, respectively, where *t* = 0 is defined as the time when the dragonfly’s body enters the camera field of vision, and the positional coordinate of the dragonfly at this time is set as (0,0,0). Under these two conditions, *η* and *ε* fluctuated periodically with time, and the fluctuation period was roughly the same as the dragonfly’s flapping period. For LD1, the average *η* and *ε* were 67.5° and 65.2°, respectively. For SD1, the average *η* and *ε* were 22.4° and 13.5°, respectively. The displacement of the center of mass in the absolute coordinate system during the whole climbing flight process can be obtained by three-dimensional reconstruction of the photo sequences of LAC and SAC. As the yaw angle during the whole climb was almost zero, the displacement along the Z direction was negligible, and the kinematics of the center of mass during LAC and SAC are recorded in Figures 4C, D. For LD1, the displacement was about 0.067 m along the *X* direction and 0.161 m along the Y direction during the whole process. For SD1, the displacement was about 0.107 m in the *X* direction and 0.044 m in the Y direction. The velocity of the center of mass during climbing was obtained by taking the derivative of displacement with time in Figures 4E, F. After low-pass filtering the centroid velocity, it can be seen that in both cases, the centroid velocities along the X and Y directions fluctuated periodically with time, and the fluctuation period was similar to the flapping period of a dragonfly. For LD1, the average climbing speed was 1.13 m/s, the *X*-direction component was 0.28 m/s, and the Y-direction component was 1.10 m/s. For SD1, the average climbing speed was 1.00 m/s, the *X*-direction component is 0.86 m/s, and the Y-direction component was 0.50 m/s. The kinematics of the body posture were similar in other groups of LAC (LD2 and LD3) to those of LD1. The same phenomenon was observed during SAC. The average velocity was obtained by using the least square method. During LAC, the average acceleration in the horizontal direction (*X* direction) and vertical direction (Y direction) were −1.05 m/s^2^ and 1.02 m/s^2^, respectively. During SAC, the average horizontal acceleration and the average vertical acceleration were 0.26 m/s^2^ and 0.96 m/s^2^, respectively.

#### Kinematic Parameters of Wing Flapping During LAC and SAC

**FIGURE 5:**
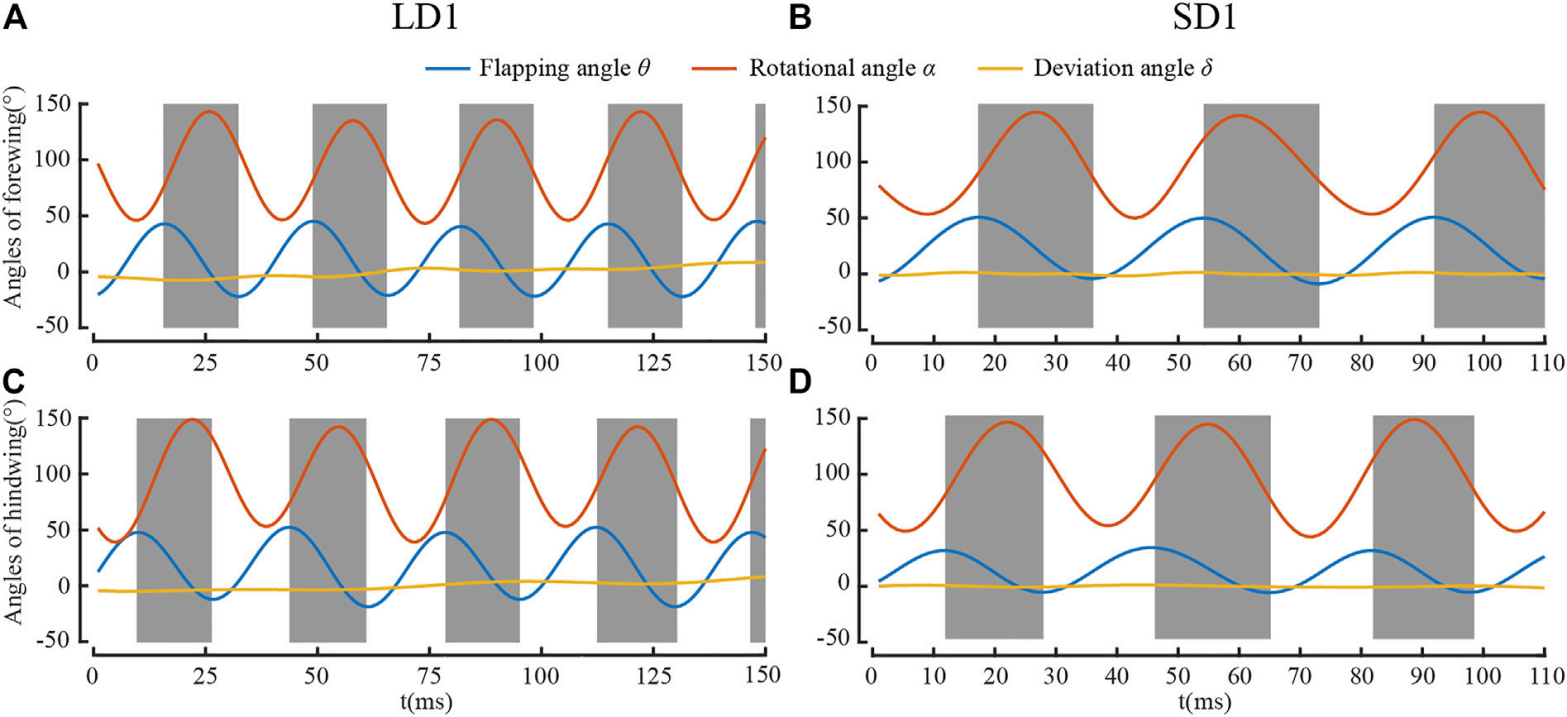
Euler angles for **(A)** forewing during LAC; **(B)** forewing for SAC; **(C)** hind wing for LAC; and **(D)** hind wing for SAC.

Figure 5 shows the time courses of the Euler angles of the forewing and the hind wing for LD1 and SD1. It can be seen that the Euler angles of the wings fluctuated periodically, and the fluctuation frequency was approximately the frequency of flapping. It is widely accepted that the positional angle *θ*, rotational angle *α*, and deviation angle *δ* can be well represented by the Fourier expansion series, including higher harmonics as follows (Azuma et al., 1985; Azuma and Watanabe, 1988):

$$A\left(t\right)=A_{0}+\sum_{n=1}^{3}\left(a_{n}\cos\left(nwt\right)+b_{n}\cos\left(nwt\right)\right),$$ (3)

where A(t) is the time course of the Euler angle, $A_{0}$ is the initial Euler angle, and $a_{n}$ , $b_{n}$ , and w are the parameters of the Fourier expansion series. The detailed kinematic parameters of the Euler angles for LD1 and SD1 are shown in Table 2.

**TABLE 2:** Detailed kinematic parameters of the Euler angles for LD1 and SD1.

	LD1								SD1							
	$A_{0}$	ω	$a_{n}$			$b_{n}$			$A_{0}$	ω	$a_{n}$			$b_{n}$		
			$a_{1}$	$a_{2}$	$a_{3}$	$b_{1}$	$b_{2}$	$b_{3}$			$a_{1}$	$a_{2}$	$a_{3}$	$b_{1}$	$b_{2}$	$b_{3}$
Forewing																
θ	17.03	95.24	0.36	−32.40	−0.18	−0.91	3.15	−0.15	21.99	56.38	0.55	−0.15	−27.75	1.15	−1.67	5.77
α	91.70	65.20	−0.32	−1.52	15.46	3.54	−0.68	−43.93	97.09	86.37	−1.77	−13.31	2.64	−5.15	−42.52	7.78
δ	1.01	28.88	−1.44	−1.34	0	−3.20	−1.07	0	−0.01	169.4	−0.95	0.06	−0.01	0.11	−0.69	0.01
Hind wing																
θ	17.33	91.71	−4.00	−6.90	0	0.29	32.04	0	13.99	89.84	−2.39	−8.42	−1.67	−1.91	17.27	−0.73
α	95.78	94.07	−7.73	−29.39	0	−0.27	−40.04	0	98.03	62.82	0.29	1.33	−27.41	2.38	−3.44	−40.48
δ	1.35	30.72	−2.02	−1.49	−1.98	−5.23	−2.14	1.20	−0.89	39.12	−0.35	0.80	1.10	1.77	0.83	−0.59

It can be seen from Table 1 and Table 2 that the kinematic parameters of wings during LAC and SAC were significantly different. *λ* of LAC (77°) was smaller than that of SAC (102°). *θ*_mean_ of the forewing (65°) and the hind wing (66°) were similar in LAC, while *θ*_mean_ of the forewing in SAC (64°) was much larger than that of the hind wing (36°). Average *δ* of the forewing and the hind wing in both cases was less than 2°.

This section introduces the kinematic pattern of dragonflies’ bodies and wings in detail during LAC and SAC by analyzing the climbing photo sequences taken in the experiment. The results show that the climbing angle *η* and wing kinematics are related. With the increase in *η*, *λ* decreased and *θ*_mean_ of the hind wing increased, while *θ*_mean_ of the forewing remained almost unchanged. To analyze the aerodynamic influence of the variations of kinematic parameters in different climbing states, the aerodynamic performance of wing flapping in these two climbing statuses will be explored using numerical simulation in *Definition of Kinematic Parameters*.

### Numerical Simulation Results of Dragonfly Climbing

The geometric parameters and kinematic parameters of LD1 and SD1 were extracted from the aforementioned experimental observations, and the aerodynamic performance was simulated using numerical simulation tools. The instantaneous lift and thrust generated during flight of LD1 and SD1 were obtained by integrating the pressure and shear stress of each node on the wing surface. Based on the aerodynamic torque and kinematic laws of the wings, the instantaneous aerodynamic power was obtained. Since the wing flap was considered to be symmetric during flight, the aerodynamic force and flow field generated by the left and right wings can be regarded as identical. Therefore, the left forewing and the left hind wing were selected for flow field analysis.

**FIGURE 6:**
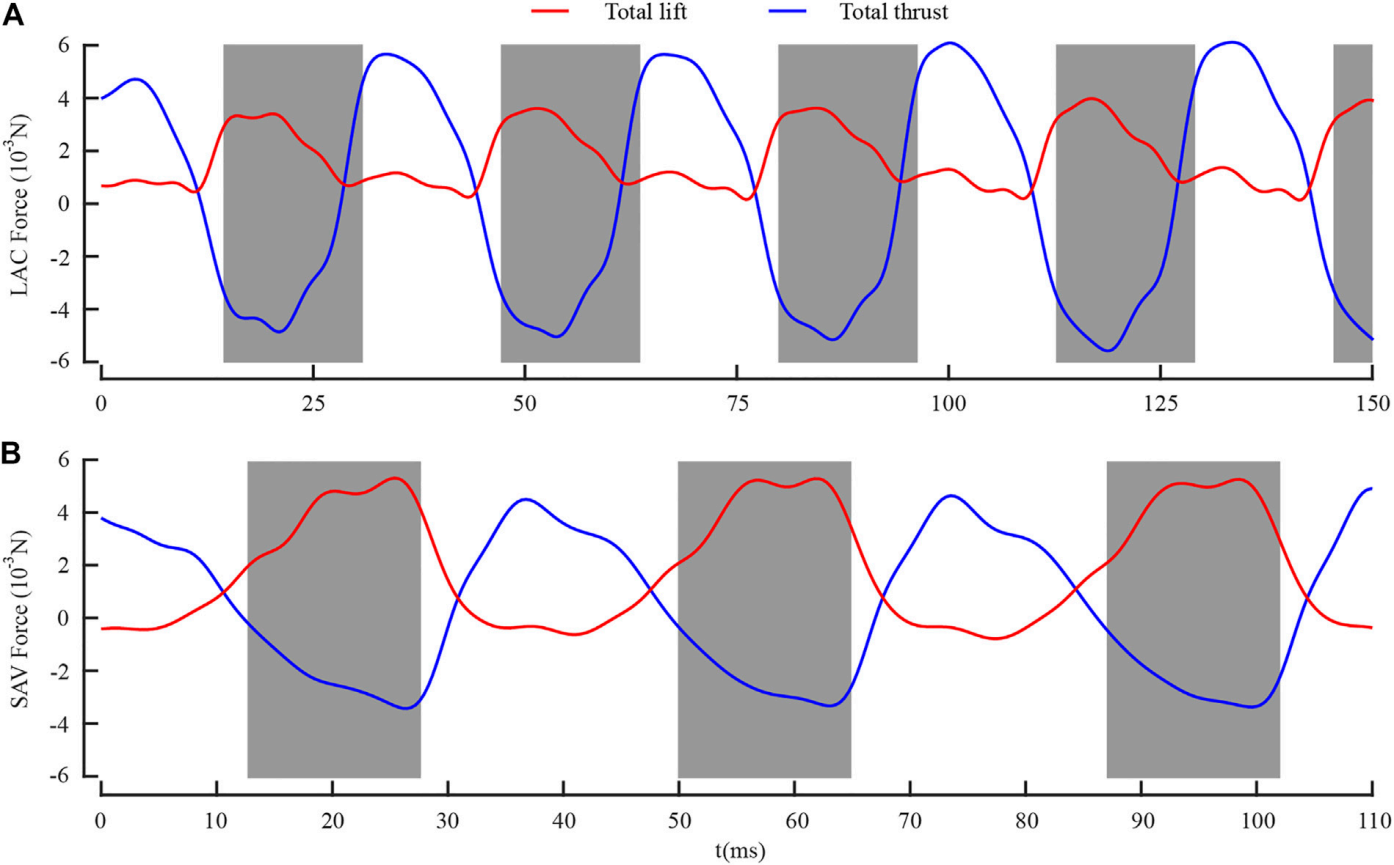
Time courses of **(A)** lift and thrust during LAC; **(B)** lift and thrust during SAC.

Figure 6 shows the total lift and thrust generated by the forewing and the hind wing during the whole LAC and SAC. The gray boxes represent the downstroke of the forewing, and *t* = 0 represents the time when the dragonfly’s body enters the camera field of vision. As shown in Figure 6A, during LAC, the total lift was positive in both the downstroke and the upstroke, while the total thrust was negative in downstroke and positive in upstroke. The maximum lift (4.08 mN) was generated in the mid-downstroke, and the maximum thrust (6.27 mN) was generated in the mid-upstroke. The cycle-averaged lift *L*_mean_ and thrust *T*_mean_ were 1.90 and 0.07 mN, respectively. As shown in Figure 6B, during SAC, the total lift was positive during the downstroke and negative during the upstroke. The maximum lift (5.29 mN) was generated in the mid-downstroke, and the maximum thrust (4.79 mN) was generated in the mid-upstroke. The *L*_mean_ and *T*_mean_ were 1.83 and 0.38 mN, respectively.

When the weight of LD1 was 345 mg, the angle between the net force and the horizontal plane was 67.6° and the average vertical acceleration was 1.00 m/s^2^. When the weight of SD1 was 335 mg, the angle between the net force and the horizontal plane was 22.3° and the average vertical acceleration was 0.94 m/s^2^. The relative differences between the direction of net force and the average vertical acceleration were within ±3%. Therefore, it can be concluded that the numerical simulation results were consistent with the experimental observations.

## Discussion

### Comparison of Aerodynamic Forces During LAC and SAC

**FIGURE 7:**
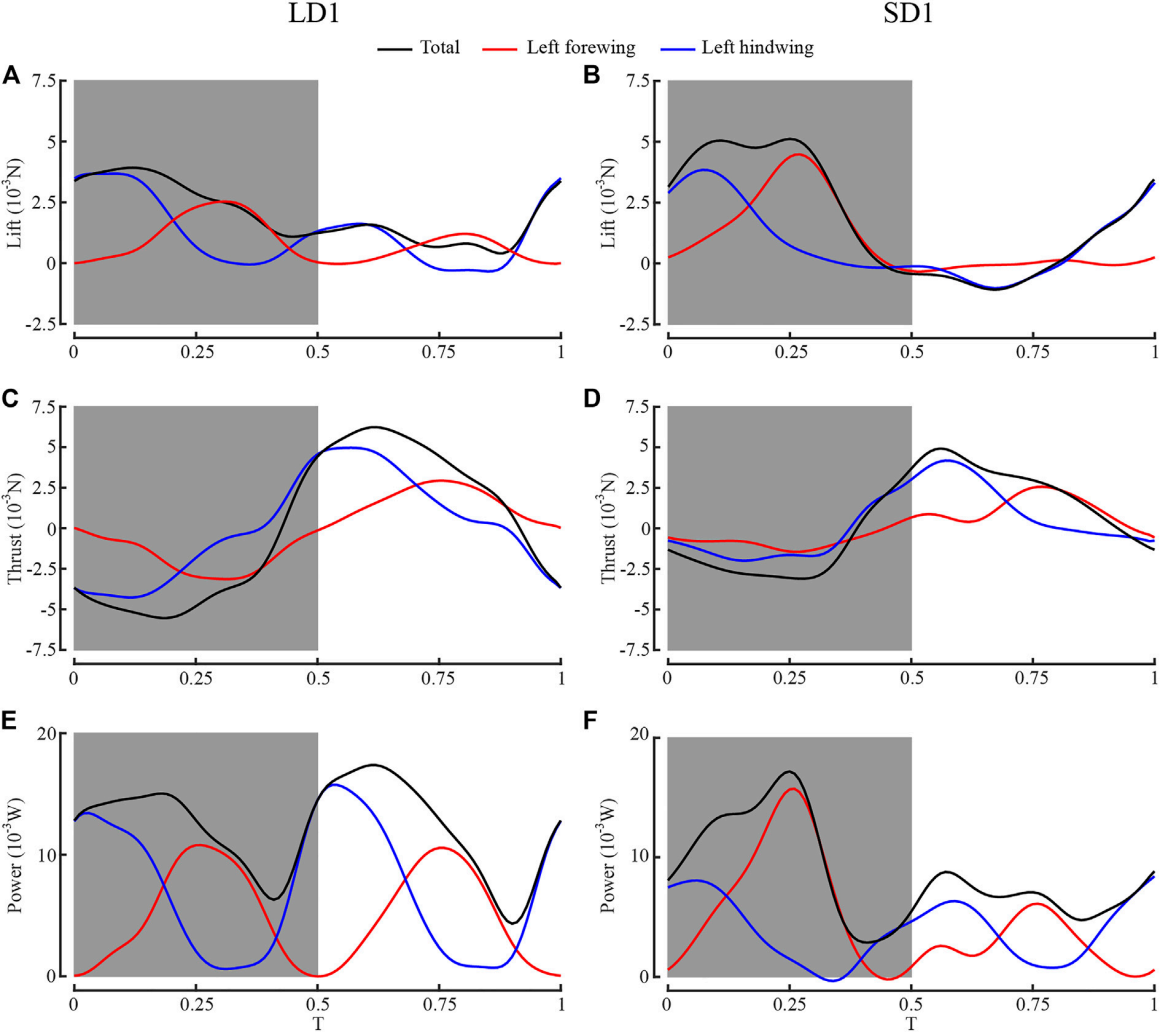
Comparison of aerodynamic performance between left forewing, left hind wing, and both wings. **(A)** Lift of LD1; **(B)** lift of SD1; **(C)** thrust of LD1; **(D)** thrust of SD1; **(E)** power of LD1; **(F)** power of SD1.

Figure 7 shows the time courses of the lift, thrust, and aerodynamic power of the left forewing, the left hind wing, and both wings during one wingbeat of LAC and SAC. The gray boxes represent the downstroke of the forewing, and *T* = 0 represents the start time of the forewing downstroke. As can be seen from Figure 7A, during LAC, the lift peak appeared in the mid-downstroke of the wing. The lift peak of the forewing appeared at *T* = 0.31, and lift peak of the hind wing appeared at *T* = 0.06 due to the phase difference. As can be seen from Figure 7C, the thrust peak appeared in the mid-upstroke of the wing. The thrust peak of the forewing at *T* = 0.76, and thrust peak of the hind wing at *T* = 0.57. The forewing contributed 40% of the whole lift, whereas the hind wing accounted for 60%. The hind wing’s lift peak (3.58 mN) and thrust peak (4.98 mN) were larger than the forewing’s lift peak (2.44 mN) and thrust peak (2.95 mN).

Similarly, during SAC, the lift peak of the forewing and the hind wing appeared at *T* = 0.27 and *T* = 0.07, respectively, and the thrust peak of the forewing and the hind wing appeared at *T* = 0.77 and *T* = 0.57, respectively. The forewing contributed 53% of the whole lift, whereas the hind wing accounted for 47%. The lift peak of the hind wing (3.87 mN) was smaller than that of the forewing (4.51 mN), and the thrust peak of the hind wing (4.11 mN) was larger than that of the forewing (2.50 mN).

In LAC, the hind wing generated more lift because *θ*_mean_ of the forewing (65°) and the hind wing (63°) was almost identical, and the surface area of the hind wing (383 mm^2^) was larger than that of the forewing (336 mm^2^), while in SAC, the hind wing generated less lift because *θ*_mean_ of the hind wing (36°) was smaller than that of the forewing (62°). During *T* = 0.07–0.31, both the forewing and the hind wing were in the middle of the downstroke and produced large lift. During *T* = 0.57–0.77, both the forewing and the hind wing were in the middle of the upstroke and produced large thrust. Therefore, dragonflies produced large aerodynamic forces in these two periods, which resulted in the peaks of total aerodynamic power in the corresponding periods, as shown in Figures 7E,F.

**FIGURE 8:**
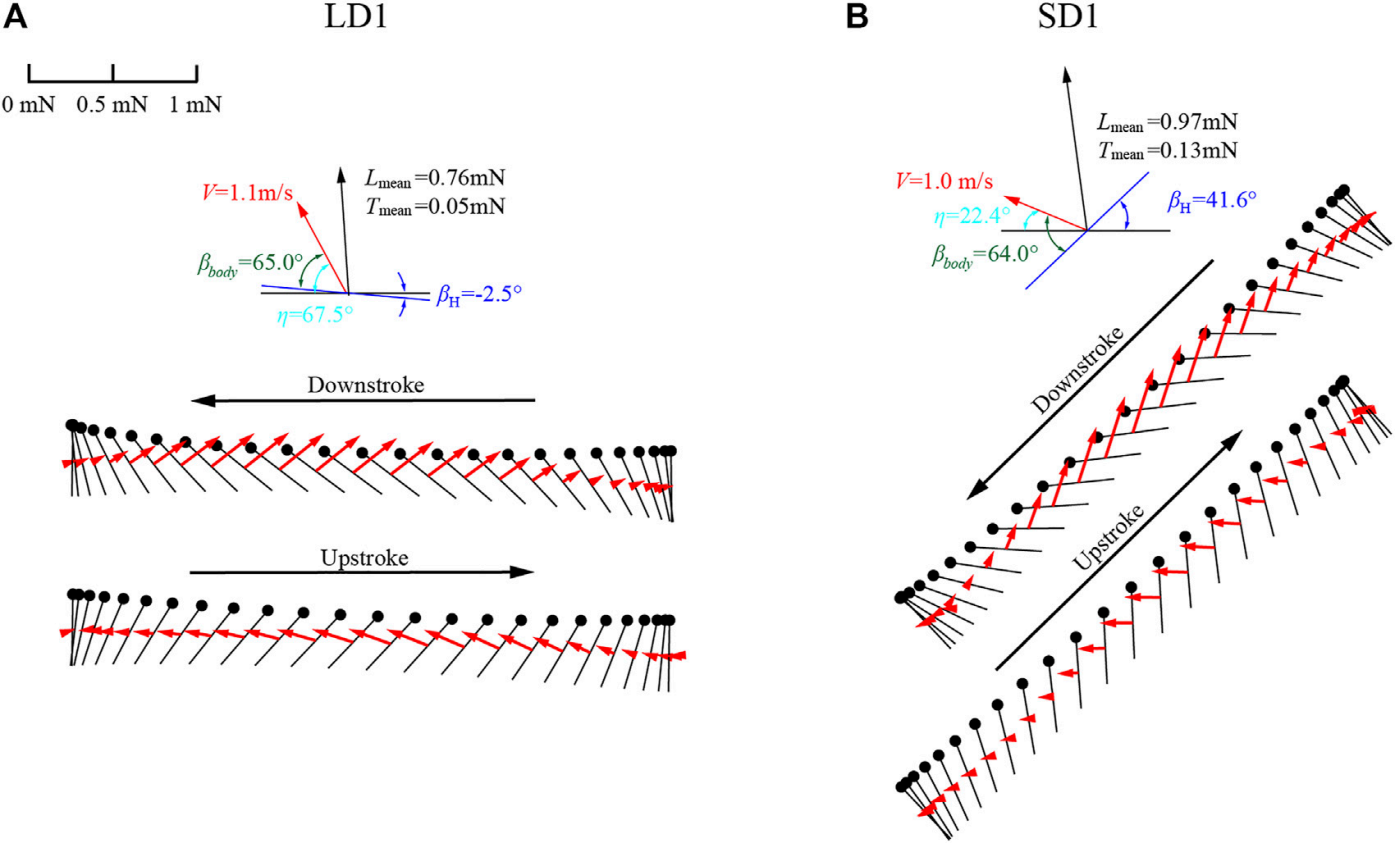
Diagram of wing motion and aerodynamic force vectors during **(A)** LAC and **(B)** SAC.

In this experiment, the angle between the flapping plane and the body axis *β*_*body*_ was almost unchanged during different climbing statuses, which is similar to the results of Azuma et al. (1985). As can be seen in Table 1, during LAC, *β*_*body*_ of the forewing and the hind wing was 65° and 66°, respectively; during SAC, *β*_*body*_ of the forewing and the hind wing was 64° and 67°, respectively. As shown in Figure 8, the diagram of wing motion and aerodynamic force vectors of the forewing during LAC and SAC were taken as examples. As *η* = *β*_*body*_-*β*_H_ and *β*_*body*_ was almost unchanged, the large difference of *η* between LAC and SAC (67.5° and 22.4°) resulted in the significant difference in *β*_H_ (−2.5° and 41.6°). Therefore, to get enough lift to counteract gravity, dragonflies employed different wing kinematics during LAC and SAC, which was the main reason for the different aerodynamic forces between the two statuses. Since the flapping plane during LAC was approximately parallel to the horizontal plane, the lift generated by the wing was positive during the downstroke and upstroke. The forewing flaps forward in the downstroke and flaps backward in the upstroke, resulting in negative thrust and positive thrust, respectively. During SAC, *β*_H_ of the forewing was 41.6°, which causes a significant vertical upward displacement of the wing during the upstroke, resulting in negative lift during the early upstroke. Therefore, when climbing with different *η*, the law of lift generated by wings will change due to the variation of *β*_H_. During LAC, the wing generates lift during both upstroke and downstroke. During SAC, the wing generates negative lift during upstroke and positive lift during downstroke.

### Comparison of Flow Fields During LAC and SAC

In order to explain the aerodynamic mechanism of the force peak and the force variation generated by wings during LAC and SAC, the three-dimensional vortex structure and two-dimensional vorticity slices along the spanwise were studied. The three-dimensional vortex structure was identified by the *Q* criterion. Based on the definition in Hunt et al. (1988), the vorticity *Q* is normalized by *Q* ^*^ *= Q/(V*_*max,2*_ */c)* ^*2*^, and *Q* ^*^ below a certain negative threshold is indicative of a vortex-dominated region. Therefore, *Q* ^*^ = −0.2 was selected as the negative threshold.

**FIGURE 9:**
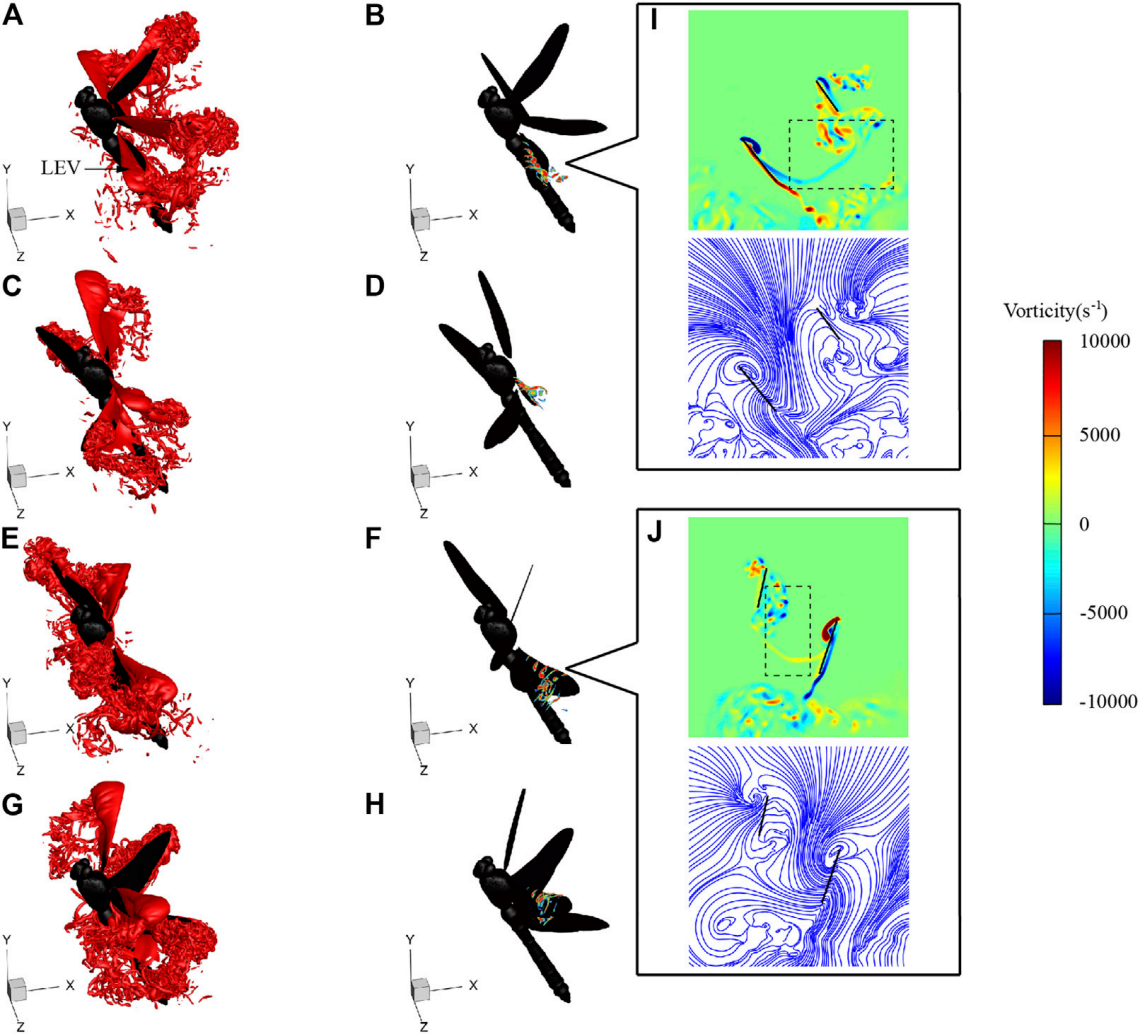
Vortex structures of LD1 at 115 ms [**(A)** and **(B)**], 118 ms [**(C)** and **(D)**], 122 ms [**(E)** and **(F)**], and 125 ms [**(G)** and **(H)**]. Vorticity contour and streamlines of the *r*_*2*_ section at 115 ms **(I)** and 122 ms **(J)**.

Figure 9 shows the flow fields at *t* = 115, 118, 122, and 125 ms during LAC, corresponding to the times of hind wing lift peak, forewing lift peak, hind wing thrust peak, and forewing thrust peak, respectively. As can be seen from Figure 9A, when t = 115 ms, the hind wing is in the mid-downstroke with an obvious leading-edge vortex (LEV) generated on the upper surface. The LEV is the main vortex structure generating lift during downstroke. The two-dimensional vorticity slices of the hind wing are shown in Figure 9B, which range from 30 to 70% along the spanwise direction from the wing root to the wing tip. It can be seen from the slices that the LEV develops from the wing root to the wing tip. The average vorticity of the hind wing LEV at *r*_2_ section is 5271 s^−1^. The radius of the second moment *r*_*2*_ is denoted by *r*_*2*_ = *∫*_*S*_ *r* ^*2*^·d*S*/*S*, where *r* is the radial distance from the wing root and *S* is the area of the wing. At *t* = 118 ms, the forewing is in the mid-downstroke, and a large and stable LEV is generated. The average vorticity of forewing LEV at *r*_2_ section is 4322 s^−1^. The smaller vorticity of the forewing is consistent with the fact that the peak lift of the forewing is smaller than that of the hind wing in *Numerical Simulation*. At this time, the hind wing is in the end of downstroke. The decrease of the flapping speed and the change of *α* led to the LEV breaking from the wingtip and losing the ability to generate lift. When *t* = 122 ms and *T* = 125 ms, the hind wing and the forewing are in the mid-upstroke, respectively, and the LEV attached to the lower surface of the wing is the main vortex structure generating thrust. When *t* = 122 ms, the average vorticity of hind wing LEV at *r*_2_ section is 4721 s^−1^; and when *t* = 125 ms, the average vorticity of forewing LEV at *r*_2_ section is 3942 s^−1^, which is consistent with the fact that the peak thrust of the forewing is smaller than that of the hind wing in *Numerical Simulation*.

**FIGURE 10:**
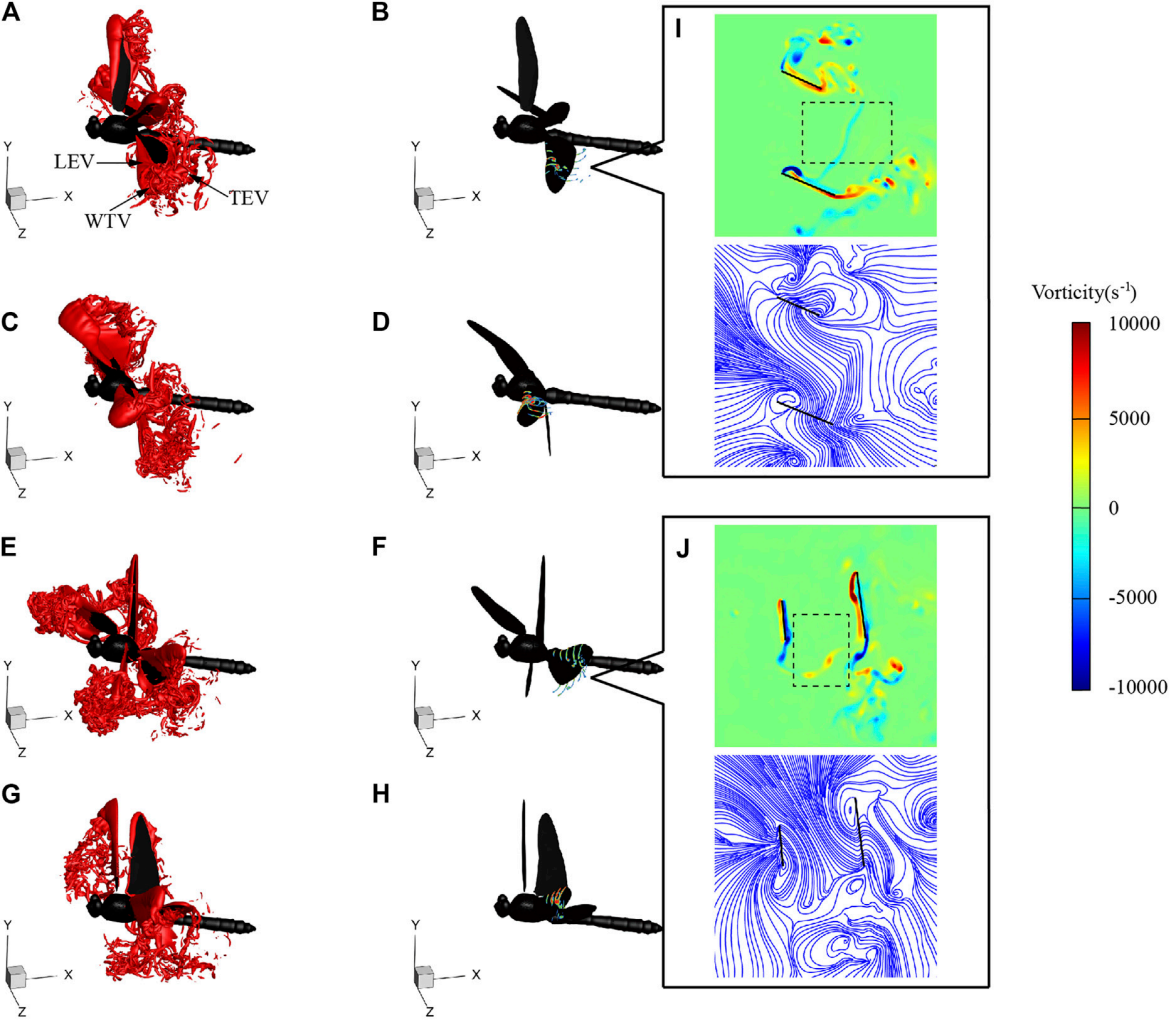
Vortex structures of SD1 at 74 ms [**(A)** and **(B)**], 76.5 ms [**(C)** and **(D)**], 80 ms [**(E)** and **(F)**], and 82.5 ms [**(G)** and **(H)**]. Vorticity contour and streamlines of the *r*_*2*_ section at 74 ms **(I)** and 80 ms **(J)**.

Figure 10 shows the flow fields at *t* = 74, 76.5, 80, and 82.5 ms during SAC, corresponding to the times of the hind wing lift peak, forewing lift peak, hind wing thrust peak, and forewing thrust peak, respectively. Similar to the flow fields of LAC, during the downstroke, LEV was generated on the leading edge of the wing and gradually developed and strengthened. With the downstroke of the wings, the vortex developed in the spanwise direction, and the LEV, wing-tip vortex (WTV) and trailing-edge vortex (TEV) formed a vortex ring, which generated most of the lift. During the upstroke, a stable LEV was generated on the lower surface of the wing. Due to the phase difference, the flapping of the forewing and the hind wing were not synchronized, which led to the movement of the wake into the vortex ring of the other wing, as shown in the black boxes of Figures 10I,J. During SAC, *θ*_mean_ of the hind wing (36°) was significantly smaller than that of the forewing (62°), resulting in a decrease in the flapping velocity and aerodynamic force of the hind wing. During downstroke, the average vorticity of hind wing LEV at *r*_2_ section at *t* = 75 ms was 2876 s^−1^, and the average vorticity of forewing LEV at *r*_2_ section at *t* = 76.5 ms was 4108 s^−1^, which means the strength of LEV generated by the hind wing decreases with *θ*_mean_. Similarly, during upstroke, the average vorticity of hind wing LEV at the *r*_2_ section at *t* = 80 ms was 3041 s^−1^, and the average vorticity of forewing LEV at the *r*_2_ section at *t* = 82.5 ms is 4214 s^−1^, which explains why the hind wing produces less thrust during upstroke.

### The Influence of Wing Kinematics on Aerodynamic Performance

The two typical climbing processes (LAC and SAC) of dragonflies have been observed and analyzed, and it was found that with the increase in *η*, *λ* increased, and *θ*_mean_ of the hind wing decreased. In this section, the influence of wing kinematics will be explained by flow field analysis.

During LAC, the flight velocity has a large component in the vertical direction. This flight status needs to overcome gravity to do work and is similar to the maneuvering flight mode like preying and escaping from enemies. In maneuvering flight, it is necessary to ensure that sufficient aerodynamic force is generated. Therefore, the large *θ*_mean_ of the hind wing is conducive to the stable output of lift and meets the aerodynamic force required for LAC. By comparing Figures 9I, 10I, it can be seen that when *η* increased, in order to ensure maneuverability, *θ*_mean_ of the hind wing increased, which increased the size and strength of LEV. Since reducing *λ* can also produce greater aerodynamic force, which has been detailed in Wang and Sun (2005) and Wang and Russell (2007), dragonflies choose to increase *θ*_mean_ of the hind wing and reduce *λ* during LAC for greater aerodynamic force.

During SAC, the velocity has a large component in the horizontal direction. The aerodynamic force generated by the wings mainly overcomes air drag. In order to reduce energy consumption and improve endurance during cruise flight like SAC, dragonflies can reduce vorticity dissipation and flow separation by adjusting wing kinematics. The vortex ring generated in the mid-downstroke of the hind wing generates a low-pressure area above the upper surface of the wing, which interacts with the wake of the forewing. As can be seen from the black boxes in Figures 9I, 10I, during the downstroke of LAC and SAC, part of the forewing wake is merged into the vortex ring of the hind wing, resulting in disturbance of the forewing wake. During SAC, the increase in *λ* enlarges the distance between the forewing and the hind wing, and the vortex ring generated by the hind wing has less influence on that of the forewing. Besides, the smaller *θ*_mean_ of the hind wing during SAC corresponds to the smaller vortex ring strength. These two factors weaken the aerodynamic disturbance of the forewing during SAC and improve the flight efficiency. The aerodynamic mechanism can also be obtained during the upstroke by comparing Figures 9J, 10J.

In order to verify the contribution of increasing *λ* and decreasing *θ*_mean_ of the hind wing to efficiency improvement, efficiencies of flapping wings during LAC and SAC were calculated. The aerodynamic power *P*_*a*_ was obtained by calculating the products of torque **M**_**a**_ and angular velocity **ω**. And the power coefficient *C*_*P*_ was calculated using

$$C_{P}=\frac{1}{0.5\rho SU_{ref}^{3}}\int_{0}^{T}\left(M_{a}\cdot \omega \right)dt,$$ (4)

where *ρ* is the fluid density and the reference velocity *U*_*ref*_ is the average velocity at the *r*_*2*_ section. The force coefficient *C*_*F*_ is calculated using

$$C_{F}=\frac{F}{0.5\rho SU_{ref}^{2}},$$ (5)

where *F* is the cycle-averaged aerodynamic force generated by the wing. The ratio of *C*_*F*_ to *C*_*P*_ is taken as a measure of efficiency, similar to the practice of Nagai and Isogai (2011) and Li and Guo (2018). The aerodynamic efficiency is 0.71 in SAC and 0.57 in LAC, indicating that the change in wing kinematics during SAC can improve the efficiency.

The influence of wing kinematics on aerodynamic performance can provide guidance for the control of MAVs. In maneuvering flight, the tandem-wing MAV can provide the required aerodynamic force by reducing the phase difference and increasing the amplitude of the positional angle of the hind wing. During cruising flight, efficiency can be improved by increasing the phase difference and decreasing the amplitude of the positional angle of the hind wing.

## Conclusion

In this study, the photo sequences of 22 dragonflies during climbing were analyzed. It was found that the climbing angles *η* were concentrated at 60°–70° (LAC) and 20°–30° (SAC). By analyzing the kinematic parameters of the body and wings, it was found that during climbing, the angle between the flapping plane and the body axis was not affected by the climbing angle. It was also found that the climbing angle *η* and wing kinematics were related. There were considerable differences in wing kinematics during climbing with different *η*, while the wing kinematics were basically unchanged during climbing with similar *η*. With the increase in the climbing angle, the flapping frequency increased, the phase difference decreased, the amplitude of the hind wing positional angle decreased, and the positional angle of the forewing remained unchanged. To meet the need for lift, dragonflies balance gravity by adjusting the kinematics of their wings during different climbing statuses, which results in different aerodynamic variations.

Through numerical simulations of LD1 and SD1, it was found that a large aerodynamic force was generated during mid-downstroke and mid-upstroke, mainly by the vortex ring composed of LEV, WTV, and TEV. During LAC, the flapping plane was almost horizontal, and positive lift was generated in both the downstroke and the upstroke. During SAC, the angle between the flapping plane and the horizontal plane was 43.6°, resulting in positive lift during the downstroke and negative lift during the upstroke; and reduction of the hind wing positional angle leads to a decrease in vortex structure strength, resulting in an aerodynamic force generated by the hind wing that was less than that of the forewing.

During SAC, the increase in *λ* and the decrease in *θ*_mean_ of the hind wing weakened the aerodynamic disturbance of the forewing by the vortex ring of the hind wing, thus improving the flight efficiency. This aerodynamic mechanism can be used to improve flight efficiency in MAV design.

## Data Availability

The original contributions presented in the study are included in the article/Supplementary Material, further inquiries can be directed to the corresponding author.
