# Supplementary material for: Kinematics and Aerodynamics of Dragonflies (Pantala flavescens, Libellulidae) in Climbing Flight
Source: Front Bioeng Biotechnol. 2022 Mar 16;10:795063. doi: 10.3389/fbioe.2022.795063 (PMC8966397; doi:10.3389/fbioe.2022.795063)
Supplement: Supplementary file 3 [file DataSheet1.PDF]

S1 Validation of the numerical method

Computational fluid dynamics simulations are conducted using the commercial solver Xflow 2019 with the lattice Boltzmann method (LBM). Unlike conventional numerical schemes based on discretization of macroscopic Navier–Stokes equations, LBM is based on microscopic models. LBM works on a spatial discretization named lattice, consisting of a Cartesian distribution of discrete points with a discrete set of velocity directions, and has successfully simulated a range of flow conditions, including porous media, human blood flow, vortex shedding, multiphase flows, droplet dynamics and turbulent flows (**Kang et al., 2016**; **Bhardwaj et al., 2018**; **Murdock et al., 2018**; **Chávez-Modena et al., 2020**; **Wang et al., 2020**).

S1.1 Two-dimensional validation

To validate the present numerical method, two typical flows of flapping wings are examined. **Wang (2000)** conducted resolved computation of two-dimensional dragonfly hovering and elucidated the vortex dynamics in lift generation. To compare with Wang's results, wing geometry, wing kinematics, air density and air viscosity from Wang's simulation are used in this method. **Figure S1** shows the two-dimensional comparison with Wang's simulation of a dragonfly hovering. **Figure S1(a)** shows the comparison of the time courses of lift coefficient (C_L) and drag coefficient (C_D) during the fourteenth cycle between Wang's simulation and present simulation. It can be seen that the force coefficients obtained by this method are in good agreement with Wang's results. **Figure S1(b)** and **Figure S1(c)** show the comparison of vorticity contours between the simulation of **Wang (2000)** and the present method. It can be seen that the flow fields near the wing surface by the present method is similar to that of **Wang (2000)**, and this method is better in wake simulation.

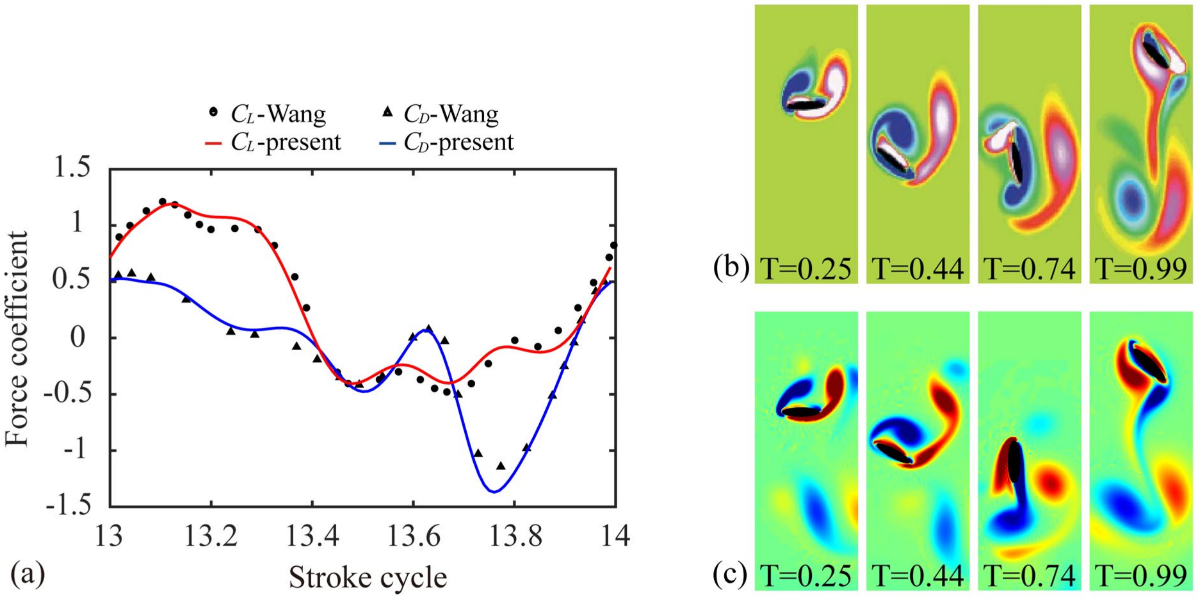

Figure S1 Two-dimensional validation of a dragonfly hovering compared with **Wang (2000)**. (a) Comparison of the force coefficient during the fourteenth cycle. (b)&(c) Comparison of the vorticity contour at the same time ((b) **Wang (2000)**, (c) present study).

S1.2 Three-dimensional validation

Dickinson et al. (1999) designed a robotic fly apparatus and drove the model wings in a tank of mineral oil to investigate the aerodynamic performance of fruitfly hovering. **Sun** and **Tang (2002)** studied the lift and power requirement for fruitfly hovering by solving the three-dimensional incompressible unsteady Navier–Stokes equations. To validate that the present method can simulate three-dimensional insect flapping, wing geometry and wing kinematics based on the study of Dickinson et al. and **Sun** and **Tang** are used in this method. **Figure S2** shows the comparison of the time courses of lift coefficient during a whole cycle for fruitfly hovering between Dickinson et al. In the model experiment, Sun and Tang in numerical simulation and present method. It can be seen that the lift coefficients obtained by this method are in good agreement with former results.

Therefore, the numerical simulation method used in this paper can accurately simulate the flow of flapping insects.

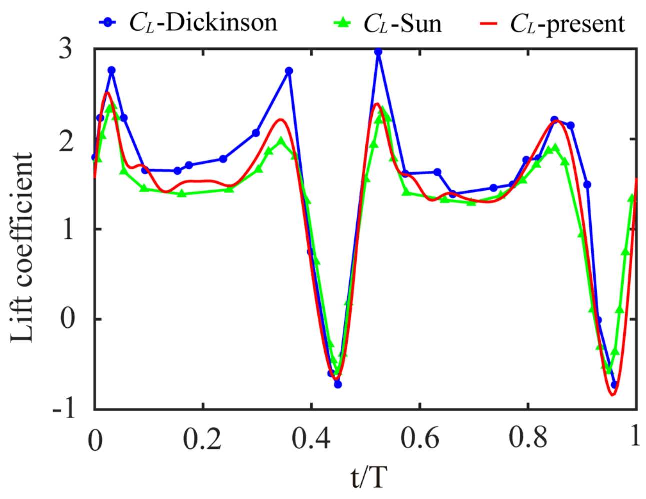

Figure S2 Three-dimensional validation of a fruitfly hovering compared with experimental and numerical data from **Dickinson et al. (1999)** and **Sun** and **Tang (2002)** respectively.

S2 Detailed settings of the simulation

S2.1 Boundary conditions

Figure S3(a) and **Figure S3(b)** show the boundary conditions and the distribution of lattices at the r_2 section, respectively. Pressure boundary conditions are applied for the inlet and outlet of the computational domain, and non-slip wall boundary conditions are used for the surfaces of wings and dragonfly body. To accurately capture the flow near the surface of the wings and the body, dynamically refined lattices based on dynamic domain topology are applied near wings and vortexes.

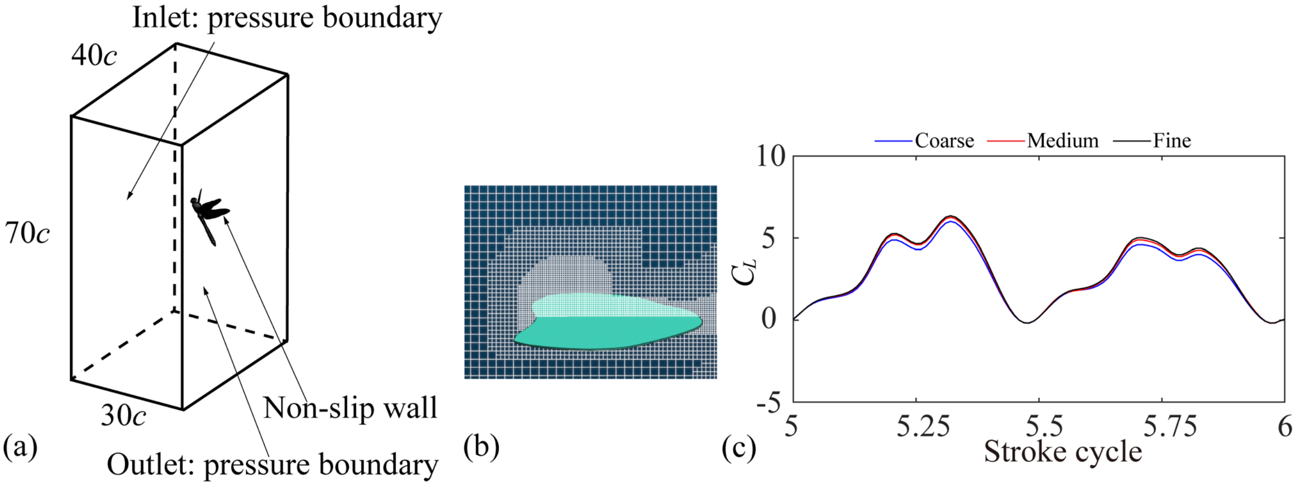

Figure S3 (a) Computational domain and boundary conditions. (b) The distribution of dynamically defined lattices at r_2 section. (c) Lift coefficient computed with coarse, medium and fine grids.

S2.2 The independence of lattice size, computational domain and time step

Before further simulation, a lattice refinement study of dragonfly hovering is conducted to determine an optimal lattice size that can provide a sufficiently accurate solution of the three-dimensional flow near the wings. The unsteady flow near the flapping wings is computed with coarse, medium and fine resolutions containing 2.05, 3.84 and 8.42 million grid points, respectively. **Figure S3(c)** shows the total lift which has been obtained on coarse, medium and fine lattice. As follows from this comparison, the medium lattice provides a lift that is nearly the same as that computed with the fine lattice. Therefore, the medium size with a target resolved scale of 0.01c is used for the cases (c is the mean chord length of the wing). The dimensions of the computational domain are set to 70c × 40c × 30c, the number of time steps per cycle is 200. When further refinements on domain and time step are applied, the variation of mean total lift is less than 0.5%.

S2.3 The settings of model wing

To make the kinematics of the body and wings of the model dragonfly consistent with the experimental observation results, the behaviour of the wings and body are configured in “Enforced” mode in Xflow. A UDF program is written by Matlab 2020 to process the kinematic data which assigns the kinematics to the dragonfly body and wings. The profile of the wing model in simulation is extracted from dragonfly wings collected in the experiment as shown in **Figure S4** with a thickness of 0.01c.

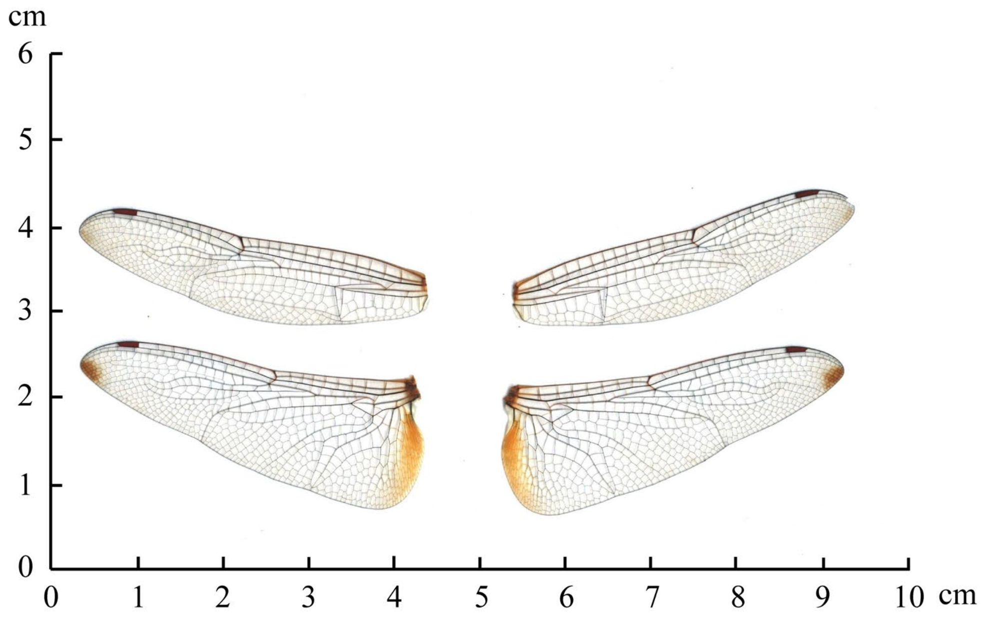

Figure S4 The profile of dragonfly wings in experiment.

S3 Experiment method

S3.1 Preparation of Animals

Pantala flavescens is a wide-distributed dragonfly of the family *Libellulidae* and it is considered to be the most widespread dragonfly on the planet. The dragonflies were collected from the pond and the main campus at Beihang University. After that, the dragonflies were stored temporarily in a cool dark storage container and then moved into the indoor laboratory.

In the laboratory, the dragonfly was first put in the refrigerator at low temperature (0-2 °C) for 1.5-2 hours. Then dragonflies were taken out and would keep in anesthesia for about 5 minutes at room temperature. In this period, the featured parameters of dragonflies (such as the weight, the morphological parameters of the wings) were measured. For three-dimensional reconstruction, some black dots were marked in the wings. All individuals were tested within 5 hours to ensure their activeness.

S3.2 Experimental facilities

As shown in **Figure S5**, a 78×78×100 cm^3 observing box was made with an iron-frame and specular glass walls to limit the flying space of the dragonflies. We also mounted two 1000 W photographic lamps for illumination compensation in the filming area. Meanwhile, two high-speed cameras (Olympus i-SPEED TR, 1000 $frames^{-1}$, shutter speed 1 ms, resolution 1280×1024 pixels) were used to film the climbing of dragonflies.

S3.3 Experimental procedure

The dragonflies were first put on the bottom platform of the observation box, which was 0.3 m below the vertical coverage area of the two cameras. Then, the video was recorded as soon as the dragonfly flew into the coverage area of the two cameras. For each dragonfly, the process of climbing was captured for 2-3 times. The total number of dragonflies tested in this paper is 22. Then select a set of climbing sequences of each dragonfly for analysis.

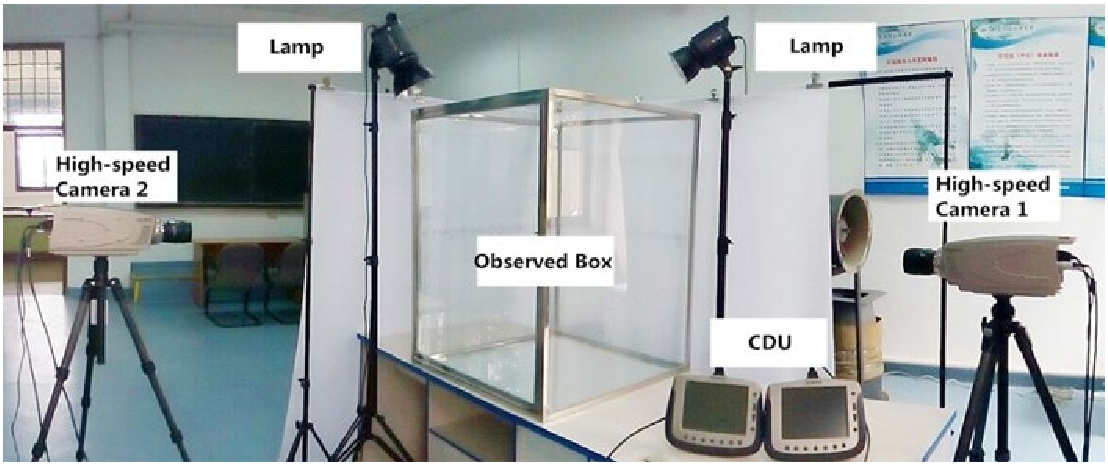

Figure S5 Experimental facilities.

Reference

Azuma, A., Azuma, S., Watanabe, I., and Furuta, T. (1985). Flight Mechanics of a Dragonfly. *J Exp Biol* 166(1). doi: 10.1213/00000539-198909000-00001.

Bhardwaj, S., Dalal, A., Biswas, G., and Mukherjee, P. (2018). Analysis of droplet dynamics in a partially obstructed confinement in a three-dimensional channel. *PHYS FLUIDS* 30(10). doi: 10.1063/1.5030738.

Chávez-Modena, M., Martínez, J.L., Cabello, J.A., and Ferrer, E. (2020). Simulations of Aerodynamic Separated Flows Using the Lattice Boltzmann Solver XFlow. *Energies* 13, 5146. doi: 10.3390/en13195146.

Dickinson, M.H., Lehmann, F.O., and Sane, S.P. (1999). Wing rotation and the aerodynamic basis of insect flight. *Science* 284(5422), 1954-1960. doi: 10.1126/science.284.5422.1954.

Galeron, G., Mazzoni, D., Amielh, M., Mattei, P.O., and Anselmet, F. (Year). "Experimental and numerical investigations of the aeroacoustics in a corrugated pipe flow", in: *Turbulence and Interactions*: Springer, Cham).

Kang, X., Tang, W., and Liu, S. (2016). Lattice Boltzmann Method for Simulating Disturbed Hemodynamic Characteristics of Blood Flow in Stenosed Human Carotid Bifurcation. *J FLUID ENG-T ASME* 138(12), 1-8. doi: 10.1115/1.4033913.

Koehler, C., Liang, Z., Gaston, Z., Wan, H., and Dong, H. (2012). 3D reconstruction and analysis of wing deformation in free-flying dragonflies. *J Exp Biol* 215(17), 3018-3027. doi: 10.1242/jeb.069005.

Lai, Y., Ma, J., and Yang, J. (2021). Flight Maneuver of a Damselfly with Phase Modulation of the Wings. *Integr Comp Biol* 61(1), 20-36. doi: 10.1093/icb/icab007.

Li, Q., Zheng, M., Pan, T., and Su, G. (2018). Experimental and Numerical Investigation on Dragonfly Wing and Body Motion during Voluntary Take-off. *Sci Rep* 8(1), 1011. doi: 10.1038/s41598-018-19237-w.

Mischiati, M., Lin, H.T., Herold, P., Imler, E., Olberg, R., and Leonardo, A. (2015). Internal models direct dragonfly interception steering. *Nature* 517(7534), 333-338. doi: 10.1038/nature14045.

Murdock, J.R., Ibrahim, A., and Yang, S. (2018). An Efficient Method of Generating and Characterizing Filter Substrates for Lattice Boltzmann Analysis. *J FLUID ENG-T ASME* 140(4). doi: 10.1115/1.4038167.

Olberg, R.M. (2012). Visual control of prey-capture flight in dragonflies. *Curr Opin Neurobiol* 22(2), 267-271. doi: 10.1016/j.conb.2011.11.015.

Olberg, R.M., Seaman, R.C., Coats, M.I., and Henry, A.F. (2007). Eye movements and target fixation during dragonfly prey-interception flights. *J Comp Physiol A Neuroethol Sens Neural Behav Physiol* 193(7), 685-693. doi: 10.1007/s00359-007-0223-0.

Olberg, R.M., Worthington, A.H., and Venator, K.R. (2000). Prey pursuit and interception in dragonflies. *J COMP PHYSIOL A* 186(2), 155-162. doi: 10.1007/s003590050015.

Peng, L., Zheng, M., Pan, T., Su, G., and Li, Q. (2021). Tandem-wing interactions on aerodynamic performance inspired by dragonfly hovering. *R Soc Open Sci* 8(8), 202275. doi: 10.1098/rsos.202275.

Saharon, D., and Luttges, M. (1989). "Dragonfly unsteady aerodynamics - The role of the wing phase relations in controlling the produced flows", in: *27th Aerospace Sciences Meeting.*).

Somps, C., and Luttges, M. (1985). Dragonfly Flight: Novel Uses of Unsteady Separated Flows. *Science* 228(4705), 1326-1329. doi: 10.1126/science.228.4705.1326.

Su, G., Dudley, R., Pan, T., Zheng, M., Peng, L., and Li, Q. (2020). Maximum aerodynamic force

production by the wandering glider dragonfly (Pantala flavescens, Libellulidae). *J Exp Biol* 223(14). doi: 10.1242/jeb.218552.

Sun, M. (2002). Lift and power requirements of hovering flight in Drosophilavirilis. *J Exp Biol* 205(16)**,** 2413-2427.

Thomas, A.L., Taylor, G.K., Srygley, R.B., Nudds, R.L., and Bomphrey, R.J. (2004). Dragonfly flight: free-flight and tethered flow visualizations reveal a diverse array of unsteady lift-generating mechanisms, controlled primarily via angle of attack. *J Exp Biol* 207(24)**,** 4299-4323. doi: 10.1242/jeb.01262.

Wakeling, J.M., and Ellington, C.P. (1997). Dragonfly flight. II. Velocities, accelerations and kinematics of flapping flight. *J Exp Biol* 200(3)**,** 557.

Wang, J., Zhang, C., Gu, S., Yang, K., Li, H., Lai, Y., et al. (2020). Enhancement of low-speed piezoelectric wind energy harvesting by bluff body shapes: Spindle-like and butterfly-like cross-sections. *AEROSP SCI TECHNOL* 103. doi: 10.1016/j.ast.2020.105898.

Wang, J.Z. (2000). Two Dimensional Mechanism for Insect Hovering. *PHYS REV LETT* 85(10)**,** 2216-2219. doi: 10.1103/PhysRevLett.85.2216.

Zou, P., Lai, Y., and Yang, J. (2019). Effects of phase lag on the hovering flight of damselfly and dragonfly. *Phys Rev E* 100(6-1)**,** 063102. doi: 10.1103/PhysRevE.100.063102.
